# The Association Between Physical Activity and Cognitive Function With Considerations by Social Risk Status

**DOI:** 10.5964/ejop.v13i4.1471

**Published:** 2017-11-30

**Authors:** Emily Frith, Paul D. Loprinzi

**Affiliations:** a Department of Health, Exercise Science and Recreation Management, The University of Mississippi, Oxford, MS, USA; Webster University Geneva, Geneva, Switzerland; University of Western Ontario, London, Canada

**Keywords:** education, elderly, executive function, exercise, poverty, minority status, stress

## Abstract

We evaluated the association between physical activity and cognitive function among a national sample of the broader U.S. adult population, with consideration by social risk. Data from the 1999-2002 National Health and Nutrition Examination Survey (NHANES) were used to identify 2031 older adults, ages 60-85. Social risk was classified by measuring four NHANES variables, namely poverty level, education, minority status, and social living status, which were graded on a scale of 0-4, with higher scores corresponding with higher social risk. The Digit Symbol Substitution Test (DSST) was used to assess cognitive function. Physical activity was assessed via a validated self-report questionnaire. After adjustments, meeting physical activity guidelines (vs not) was associated with greater cognitive function (β = 3.0, 95% CI [1.5, 4.4], p < 0.001). In this same model, social risk status was also independently associated with cognitive function. Meeting physical activity guidelines (vs. not) was not associated with higher cognitive function among those with a social risk score of of 3 (β = -0.01; 95% CI [-6.3, 6.4], p = 0.99) or a social risk score of 4 (β = -6.8, 95% CI [-15.7, 2.0], p = 0.12). In this national sample of older adults, meeting physical activity guidelines, and degree of social risk were independently associated with cognitive function. However, physical activity was not associated with cognitive function among older adults with the highest degree of social risk.

Rarely does social risk emerge as the product of a single measure of poor health (Adler, Bush, & Pantell, 2012; McEwen, 1998). Social risk is an umbrella term often defined by poverty level, education, minority status, and social living status, which interact along a continuum that defines individual susceptibility to a myriad of hazardous health outcomes (Caleyachetty et al., 2015; Loprinzi & Davis, 2016). Thus, the weight of social risk on measurable health outcomes is a function of social class, socioeconomic status, opportunities for employment, access to healthcare, safe and sanitary environments, reliable transportation, resources to initiate and sustain exercise and healthy behavior, and positive social interactions (Kuiper et al., 2015; Sutin, Stephan, Carretta, & Terracciano, 2015). In addition to poor health, social risk factors may be linked to reductions in normal cognitive function (Lynch, Kaplan, & Shema, 1997; Oakes, 1990; Sutin et al., 2015).

Disturbances in neuronal connections may be catalyzed by social inequality (Meyer-Lindenberg & Tost, 2012). Social inequality is an issue of modern relevance, as persistent racial tensions and class discrimination create significant division across and within societies worldwide (Sutin et al., 2015). Economic hardship has been shown to affect levels of depression, memory, and attentional focus among adults ages 45 and older (Lynch et al., 1997). Lack of positive social interactions are also associated with incident dementia in elderly individuals (Kuiper et al., 2015). Cognitive manifestations of class disparity have also been observed in younger populations; however, it is unclear if cognitive dysfunction is a result of stressors implicit in discrimination, or if opportunities for cognitive advancement are lacking for marginalized individuals of higher social risk (Oakes, 1990). The harmful consequences of social exclusion on cognitive dysfunction have been compared to health risks associated with low-educational attainment, depression, and physical inactivity (Kuiper et al., 2015).

Emotional processing, stress response, and salience are also negatively affected by rising social risk, and potentially contribute to symptoms of mental illness and reduced resiliency in combatting various stressors (Meyer-Lindenberg & Tost, 2012). The protective effects of exercise adherence in attenuating maladaptive management of acute and long-term stress, have been well documented (Jackson, 2013). The physiological basis of exercise-attenuated stress is influenced by hormonal changes, as well as neurotransmitter activity (Jackson, 2013), including dopamine and serotonin action on cerebral structures; especially the brain’s limbic system, which attends to emotional processing of negative and positive stimuli (Esch & Stefano, 2010). Moderate-intensity exercise is thought to induce a pleasurable, calming effect, promoting health and facilitating adequate stress management (Esch & Stefano, 2010; King, Baumann, O’Sullivan, Wilcox, & Castro, 2002).

Historically, exercise has been identified as a eu-stressor which may not only benefit physical wellness (Jackson, 2013), but may also optimize cognition (Davey, 1973) in older individuals. As social risk factors, such as economic hardship, are suggested to contribute to deleterious mental and physical functioning, and mortality risk (Lynch, Kaplan, & Shema, 1997), physical activity may be critical in protecting against these adverse health outcomes. As noted previously, both physical activity and social risk may influence cognitive function, but to our knowledge, limited research has evaluated them collectively on cognitive function, and further, whether physical activity is still associated with higher cognitive function among those with worse social risk status. Therefore, the purpose of this study was to examine the potential for physical activity and social risk status to independently associate with cognitive function among an aging population. A subsequent aim of this study was to determine the relationship between physical activity status and cognitive performance among those with higher (worse) social risk scores; that is, whether social risk status moderates the association between physical activity and cognition.

## Methods

### Study Design

The 1999-2002 NHANES data was used. The NHANES is an ongoing survey conducted by the Center for Disease Control and Prevention designed to evaluate the health status of U.S. adults through a complex, multistage, stratified clustered probability design. Further details of NHANES can be found elsewhere (http://www.cdc.gov/nchs/nhanes.htm).

### Participants

Written informed consent was obtained from all participants. Participants included 2,031 older adults (60-85 yrs) with complete data on the study variables.

### Procedure

Self-reported physical activity, objectively-measured cognitive function, and self-reported social risk NHANES data (1999-2002) from 2,031 older adult participants ages (60-85) were assessed. These relationships were determined using multivariate linear regression techniques.

#### Physical Activity

As described elsewhere (Loprinzi, 2015), participants were asked open-ended questions about participation in leisure-time physical activity over the past 30 days. Data was coded into 48 activities, including 16 sports-related activities, 14 exercise-related activities, and 18 recreational-related activities.

For each of the 48 activities where participants reported moderate or vigorous-intensity for the respective activity, they were asked to report the number of times they engaged in that activity over the past 30 days and the average duration they engaged in that activity.

For each activity, Metabolic Equivalent of Task (MET)-min-month was calculated by multiplying the number of days, by the mean duration, by the respective MET level (MET-min-month = days*duration*MET level). The MET levels for each activity are provided elsewhere. Participants engaging in 2000+ moderate-to-vigorous physical activity (MVPA) MET-Min-Month were defined as meeting physical activity guidelines. As described elsewhere (Loprinzi, 2015), this self-reported physical activity measure has demonstrated evidence of convergent validity by associating with accelerometer-assessed physical activity.

#### Cognitive Function

As describe elsewhere (Frith, Addoh, Mann, Windham, & Loprinzi, 2017), the Digit Symbol Substitution Test (DSST) was used to assess cognitive function; only NHANES participants 65+ years of age were eligible for the DSST assessment. The DSST, a component of the Wechsler Adult Intelligence Test and a test of visuospatial and motor speed-of-processing, has a considerable executive function component and is frequently used as a sensitive measure of frontal lobe executive functions (Parkin & Java, 1999; Vilkki & Holst, 1991). The DSST was used to assess participant cognitive function tasks of pairing (each digit 1-9 has a symbol it is associated with) and free recall (allowing participants to draw more figures in the limited time due to remembering pairs). Participants were asked to draw as many symbols as possible that were paired with numbers within 2 min. Following the standard scoring method, one point is given for each correctly drawn and matched symbol, and one point is subtracted for each incorrectly drawn and matched symbol, with a maximum score of 133.

#### Social Risk

Consistent with other studies (Caleyachetty et al., 2015; Loprinzi & Davis, 2016), social risk was assessed from four NHANES variables, namely poverty level, education, minority status, and social living status. For poverty level, participants were dichotomized into being above or below the poverty level, with an income-to-poverty ratio of < 1 denoting below the poverty threshold. For education, participants were dichotomized as < 12^th^ grade education or 12^th^ grade or higher education. For minority status, participants were classified as either non-Hispanic white or other (Mexican American, other Hispanic, non-Hispanic black, or other race). Lastly, for social living status, participants were classified as married/living with a partner or other (widowed, divorced, separated or never married). Based on these 4 social risk parameters, the possible range for this variable is 0-4; those with a social risk of “4” were below the poverty level, had < 12^th^ grade education, were not a non-Hispanic white, and were not married or living with a partner. Thus, a higher social risk score indicates a worse social risk level.

#### Statistical Analysis and Covariates

Multivariable linear regression analysis was used to examine the association between meeting MVPA guidelines and cognitive function (outcome variable). Analyses were computed separately across the social risk status levels and adjusted for the NHANES complex, multistage probability design. In all models, the following covariates were included: *age* (continuous; yrs), *gender*, physician-diagnosed *hypertension* (yes/no), *A1C* (continuous; %), *energy intake* (continuous; kcals), *A1C* (continuous; %); *overweight* (measured BMI ≥ 25 kg/m^2^ vs. a BMI < 25 kg/m^2^), and self-reported *smoking status* (current, former or never smoker). Statistical significance was established as *p <* 0.05.

## Results

Weighted characteristics of the study variables are shown in Table 1. Older adults with a higher social risk score engaged in less MVPA. The mean MVPA MET-min-month among those with a social risk score of 0, 1, 2, 3, and 4, respectively, was 4555.8, 2816.5, 2352.6, 2739.8, and 1979.5. Following this trend, the proportion of adults meeting MVPA guidelines across these respective social risk groups was: 49.2%, 32.2%, 25.5%, 18.9%, and 11.6%.

**Table 1:** Weighted Characteristics of the Analyzed Sample, 1999-2002 NHANES (N = 2031)

Variable	Point Estimate (SE)
DSST, mean	47.7 (0.7)
Social Risk Score, %	
0	44.3
1	33.1
2	14.0
3	6.4
4	2.2
Age, mean years	69.9 (0.3)
% Female	54.4
% Meeting MVPA guidelines	37.4
% Overweight/obese	71.1
% Physician-diagnosed hypertension	50.0
% Smoker	12.4
Energy intake, mean kcals	1790.9 (22.8)
A1C, mean %	5.8 (0.02)

*Note.* A1C = Glycosylated hemoglobin; DSST = Digit symbol substitution test; MVPA = Moderate-to-vigorous physical activity.

In a multivariable linear regression model adjusting for all covariates as well as social risk status, meeting physical activity guidelines (vs. not) was associated with greater cognitive function (β = 3.0, 95% CI [1.5, 4.4], *p <* 0.001). In this same model, social risk status was also independently associated with cognitive function, demonstrating a clear dose-response relationship; compared to those with a social risk score of 0, the adjusted β’s for those with a social risk score of 1, 2, 3, and 4, respectively, were -7.7 (95% CI [-9.6, -5.7], *p <* 0.001), -14.5 (95% CI [-16.8, -12.2], *p <* 0.001), -23.1 (95% CI [-25.8, -20.3], *p <* 0.001), and -31.0 (95% CI [-37.0, -24.9], *p <* 0.001). Thus, this model demonstrates that greater MVPA participation is independently associated with greater cognitive function, and those with a higher (worse) social risk had lower cognitive function.

Additional analyses evaluated whether meeting physical activity guidelines was associated with cognitive function across the different social risk levels. Notably, physical activity was only associated with better cognitive function among those with a lower (better) social risk score. After adjustments, meeting physical activity guidelines (vs. not) was associated with higher cognitive function among those with a social risk score of 0 (β = 2.1, 95% CI [0.13, 4.0], *p =* 0.03), a social risk score of 1 (β = 3.8, 95% CI [1.2, 6.4], *p =* 0.006), a social risk score of 2 (β = 5.1, 95% CI [2.0, 8.1], *p =* 0.002), but not among those with a social risk score of 3 (β = -0.01, 95% CI [-6.3, 6.4], *p =* 0.99) or a social risk score of 4 (β = -6.8, 95% CI [-15.7, 2.0], *p =* 0.12).

## Discussion

Previous work has demonstrated the independent influences of physical activity and social risk on cognition function (Loprinzi & Davis, 2016; Meyer-Lindenberg & Tost, 2012). Specifically, poverty has been shown to corroborate reduced physical, psychological, and cognitive functioning (Lynch, Kaplan, & Shema, 1997). To our knowledge, limited research has evaluated both their independent and combined associations in a single cohort, and further, limited research has evaluated the role of social risk as a moderator in the relationship between physical activity and cognitive function. This was the aim of our study. Both physical activity and social risk were independently associated with cognitive function; although, within our sample, physical activity did not appear to be beneficially associated with cognitive function among those with a higher (worse) social risk score. This finding underscores the importance of tailored attention to individuals with a high social risk status (i.e., minority older adults, those living alone, those with less education, and those below the poverty level). As shown in the results, physical activity promotion among those with a higher social risk score may be particularly needed as those with the highest social risk score were the least active. Increased levels of inactivity could explain the divergent results between elderly participants classified as scoring 0-2 on our index of social risk, compared to participants scoring higher. Absence of an association between physical activity and cognitive function among those characterized as having higher social risk, strengthens our suggestion that physical activity promotion must target marginalized and underserved populations.

Although utilization of a cross-sectional design was appropriate for our analysis of this national sample, cross-sectional and experimental research has provided convincing evidence on the neuroprotective benefits of physical activity on cognition (Erickson, Hillman, & Kramer, 2015; Lautenschlager et al., 2008). The breadth of these benefits is substantive, with improvements in late-life cognition spanning an array of cognitive domains (Smith et al., 2010); particularly involving tests of executive function (Colcombe & Kramer, 2003). While future experimental research should aim to investigate the effects of physical activity on an array of cognitive tests, the DSST was an appropriate measure of executive function among individuals within this sample. Brain imaging highlights improvements in white matter integrity and gray matter volume in line with physical activity participation. Specifically, the frontal cortex and hippocampus are gray matter structures subject to positive modifications in total volume as a result of regular exercise engagement (Erickson, Hillman, & Kramer, 2015).

Brain imaging research also implicates stress as a catalyst for structural changes in cortical, limbic, and hippocampal structures (Musazzi, Treccani, & Popoli, 2015). Stress is a contributing factor to depression and anxiety disorders, which are prevalent among individuals with higher social risk (Meyer-Lindenberg & Tost, 2012). Morphological changes include dendritic atrophy and remodeling of neurons within the prefrontal cortex, which is a cognitive structure necessary for learning, working memory, and executive function (Musazzi et al., 2015; Stuss & Knight, 2002). It may be impossible to accurately pinpoint stress levels that exceed abilities for successful coping and adaptation. However, the myriad of stressors described for those with elevated social risk, may exert a profoundly multiplicative impact on physical and mental fortitude in the older population.

We must also consider the likelihood of reverse causation as a limitation of our study. Our findings show an inverse association between physical activity levels and social risk across four NHANES variables. However, social risk may also be an antecedent to physical activity initiation and maintenance. Minority adults may not feel comfortable engaging in physical activity if social isolation is a concern. Cultural differences may also play a role in hedonistic and affective responses to physical activities characteristic of American societies. Older adults, living alone, may lack sufficient self-efficacy related to physical activity, and may feel unsafe or insecure when attempting to be active in solitude. Fall risk among elderly individuals is another concern that may influence the decisions of older adults to be physically active. Barriers also include securing reliable transit to and from safe spaces for exercising, as well as the increased proportion of elderly men and women who do not live in close proximity to environments conducive to exercise (Schutzer & Graves, 2004). Individuals subject to inadequate educational opportunities, are likely to receive a paucity of credible education on the benefits of physical activity and lifetime wellness. Older adults with a lower socioeconomic status may also be less likely to have access to exercise facilities, or equipment, such as proper walking shoes.

In conclusion, results of this study show an inverse relationship between physical activity participation and social risk. A significant linear association was also observed between cognitive dysfunction and social risk. Participants who met MVPA guidelines were less likely to score higher (worse) across social risk variables, including poverty level, education, minority status, and social living status. However, the beneficence of physical activity does not appear to attenuate social risk among individuals with the highest social risk scores. Therefore, physical activity is instrumental in preventative care. Social risk scores are not static, meaning that the degree of social risk could potentially be augmented as a function of time spent facing a variety of stressors, specifically low socioeconomic status, opportunities for personal growth and societal inclusion, and access to resources for attaining healthcare and observing healthy behaviors. Stress is thought to be a contributing factor to cognitive dysfunction among those with greater predicted social risk. Understanding the deleterious association between excessive amounts of distress and neural remodeling is of critical importance for individuals, and professionals in the health industry. Optimal education and strategies for stress management are necessary aims for maintenance of cognitive function, as well as learning to develop resilience despite adversities inherent to social risk. In a society where social classes are distinct and often pejorative, social risk will remain a public health concern. Although equitable societal reform may be suggestive of an idealistic prospective, there is much that can be accomplished in the public health sector to increase opportunities for individuals to thrive. Future research should examine independent effects of social risk within the built environment on physical activity and cognition among older populations, as well as constructing experimental research designs to more comprehensively assess these multifaceted relationships.
